# Assessing the Need for Pediatric Palliative Care in the Six Arab Gulf Cooperation Council Countries

**DOI:** 10.1089/pmr.2022.0037

**Published:** 2023-03-24

**Authors:** Qutaibah Alotaibi, Manjiri Dighe

**Affiliations:** Pediatric Department, Aladan Hospital, Hadiya, Kuwait.

**Keywords:** Gulf Cooperation Council, incidence, pediatric palliative care, prevalence

## Abstract

**Background:**

Palliative care is an essential element of universal health coverage. However, palliative care services, particularly pediatric palliative care (PPC) services, are still inadequately developed in many countries, not least members of the Gulf Cooperation Council (GCC) (Bahrain, Kuwait, Oman, Qatar, Saudi Arabia, and the United Arab Emirates). Advocating for palliative care services requires data-driven estimates of the number of patients needing these services.

**Objective:**

To estimate the number of children living with life-threatening illnesses in the GCC countries requiring specialist and/or generalist palliative care service provision.

**Method:**

Descriptive analysis of published cross-sectional epidemiological data. Subjects were from general and age-specific populations from individual GCC countries. The quantitative data on child population and mortality were collected from 2019 primary and secondary data sources. The need for PPC was estimated using mortality, incidence, and prevalence data from the Institute for Health Metrics and the Global Cancer Observatory.

**Results:**

Our conservative analysis revealed that just under 22,000 children needed PPC in GCC countries in 2019, a minimum of 17.5 for every 10,000 children.

**Discussion:**

There is a significant need for PPC services, suggesting that the medical needs of the pediatric population are currently not being fully met. Nationwide PPC services are essential to improve the quality of life of thousands of children in GCC countries by changing policies, professional education, and providing funding to palliative programs. To our best knowledge, this is the first study to highlight the clear and urgent need for the development of PPC services in the GCC countries.

## Introduction

There is growing international recognition of the importance of pediatric palliative care (PPC) and the need to integrate it into standard medical care. PPC provides relief from the physical, social, emotional, and spiritual suffering of children with life-threatening illnesses (LTIs). The World Health Organization (WHO) values palliative care for children and considers its inclusion in health services as “an ethical responsibility of health systems,” regardless of limitations in resources.^1^ However, there is still only very limited PPC provision in Gulf Cooperation Council (GCC) countries and, where available, it has yet to be fully integrated into health systems.^1–4^

The GCC countries are Bahrain, Kuwait, Oman, Qatar, Saudi Arabia, and the United Arab Emirates. Their total estimated population in 2019 was 57.4 million, with Saudi Arabia having the largest population (59%) and Bahrain the smallest (2.6%).^5^ About a fifth of the total GCC population are children, and this population is increasing by 1.4 million each year.^5^ GCC countries are classified as “high-income” countries by The World Bank.^6^ GCC health care expenditure is around 2.6%–4.7% of gross domestic product, and from the benefit of oil revenues these countries have achieved good health outcomes through universal access to health services funded fully by their governments.^7,8^

The private health care sector (private hospitals and clinics) currently provides a small proportion of medical services to patients who wish to pay out of pocket.^7,8^ However, the private health care sector has recently expanded to cover gaps in the growing health care needs of primary and specialty care services, with GCC governments introducing national health insurance schemes as a cost-containment measure.^7,8^

According to the WHO, GCC countries share health system disadvantages including disorganization and weak policy formulation, regulation, and analysis, which also negatively affect private sector quality assurance.^8^ Furthermore, with regard to palliative care, none of the six countries currently provide palliative care services at a preliminary or advanced stage of integration into mainstream services, with most available palliative care provided primarily for adults.^1–4^ According to The International Children's Palliative Care Network (ICPCN), only Kuwait and Saudi Arabia evidence localized palliative care provision for children, and, to our best knowledge, there are currently only four PPC-trained doctors in those two countries.^4^ The other four countries have no known specialized PPC services.^4^

To facilitate future development of PPC provision, here we estimated the number of children living with LTIs in the GCC countries. Since there is no registry nor formal statistics of diagnoses in GCC countries, we relied on estimates from the Institute for Health Metrics and Evaluation (IHME), an independent global health statistics research institute at the University of Washington, to assess the incidence and prevalence of diseases that cause LTI.^9^

## Methods

### Data sources

Data for the general population, age-specific population, and birth and death rates were estimated from (1) the official statistics agencies from individual GCC countries, from which gross age-specific population, birth, and death statistics were gathered;^10–15^ (2) the Statistical Centre for the GCC countries (GCC-Stat), which serves as a common official pool of statistical data for GCC members, from which population comparisons, percentages, and expected growth were obtained;^5^ and (3) UN population data, which were used where details for age-specific statistics were not available from a country's official statistics agencies, like the United Arab Emirates.^16^

The incidence, prevalence, and mortality of diseases that cause LTIs were collected from the IHME.^9^ Data were from 2019, since this was the most recent year that complete data were available from the sources. The data predate deaths that may have been attributed to the COVID-19 pandemic.

### Definitions

Here, the term “children” refers to neonates, infants, and children up to 14 years of age, as most countries in the GCC provide pediatric services to children around 14 years before transferring them to adult services. Regarding eligibility for PPC services, we used the criteria outlined in the fourth edition of “A Guide to Children's Palliative Care” published by “Together for Short Lives” (TFSL), a charitable organization formed after the Association for Children's Palliative Care merged with Children's Hospices UK.^17^

This publication is endorsed by The Royal College of Paediatrics and Child Health UK, ICPCN, and the European Association for Palliative Care.^17^ Therefore, children's LTIs eligible for PPC were divided into four groups:^17^ (1) life-threatening conditions for which curative treatment may be feasible but can fail; (2) conditions where premature death is inevitable; (3) progressive conditions without curative treatment options; and (4) irreversible but nonprogressive conditions causing severe disability leading to susceptibility to health complications and likelihood of premature death.

### Study design

Using the IHME's “Global Burden of Disease” search engine, under causes of death, we used the search criteria of measures (deaths, incidence, and prevalence), locations (the six countries), age groups from early neonatal to 14 years, and the year 2019.^9^ Data were input into Apple Numbers spreadsheet software. Finally, we excluded sudden deaths and nondisease causes of death such as suicide and accidents.

Pediatric deaths attributable to known diseases or medical conditions were identified and grouped into five categories: neonatal conditions, infectious diseases, neoplastic diseases, congenital (including genetic) disorders, and other diseases. We then evaluated the different diseases and allocated them to the four groups described in the TFSL guide. Finally, an evidence-based approach was used to estimate children living with those fatal diseases (see Table 1). Data were analyzed and tabulated in Tables 2 and 3.

**Table 1.** Collected Data and the Methodology Used to Estimate Pediatric Palliative Care Needs

Mortality causes	Methodology
Neonatal conditions	• Included mortality estimates from prematurity, encephalopathy, neonatal hemolytic diseases, and other neonatal conditions. • Excluded neonatal sepsis due to its acuity and high reversibility • Added all extreme prematurity (estimation: 4.1% of premature births)^18^ because they fit the first group of TFSL classifications.^17^
Infectious diseases	• Included mortality estimates from HIV, tuberculosis, meningitis, encephalitis, tetanus, measles, diphtheria, pertussis, tetanus, acute hepatitis, enteric, tropical, and “other unspecified infections.”^9^ • Excluded other infections not specified above due to the high reversibility and acuity.
Neoplastic diseases	• Included mortality estimates from malignant cancers • Added the following:^19–24^ • Malignant cancer incidence estimates to represent children receiving first therapy. • 25% of malignant cancer prevalence estimates to represent children with advanced cancer.^25^
Congenital birth defects	• Include mortality estimates of organ defects, such as neural tube defects or cardiac anomalies, and genetic conditions. • Added 45%^26,27^ of congenital heart diseases incidence to represent single ventricle and single ventricle pathway defects, severe congenital heart anomalies (excluding single ventricle defects), and critical malformations of the great vessels, which fit the definition of the first group of TFSL classifications.^15,28^
Other diseases	• Included mortality estimates from all other systemic diseases not included in the mentioned categories. • Added 25% of cerebral palsy patients (Estimation: 0.5:1000 children),^29^ who fit the definition of the fourth group of TFSL classifications.^17,30^

TFSL, Together for Short Lives.

**Table 2.** Distribution Estimates of Causes of Death in the Gulf Cooperation Council Countries in 2019

Causes	Infection	Neoplasms	Neonatal	Congenital	Other diseases
Bahrain	11	7	32	31	21
Kuwait	58	28	200	245	49
Oman	136	33	318	220	85
Qatar	22	12	80	89	22
Saudi Arabia	635	124	736	1055	209
United Arab Emirates	54	37	76	147	51
Total	916	241	1442	1787	437

Values represent number of deaths from each respective country.

**Table 3.** Estimated Number of Children Living with Life-Threatening Illnesses in Gulf Cooperation Council Countries in 2019, and Ratios per 10,000 Children

Country	Pediatric population	Congenital diseases	Neonatal	Infection	Neoplasm	Other diseases	Prevalence of PPC need	Rate in 10,000
Bahrain	271,265	146	113	5	35	157	456	16.8
Kuwait	902,931	622	433	14	147	500	1716	19
Oman	1,095,508	741	668	66	192	633	2300	21
Qatar	398,435	280	203	12	51	221	767	19.3
United Arab Emirates	1,447,414	781	487	35	233	775	2311	16
Saudi Arabia	8,389,963	5051	3262	211	1409	4404	14,337	17.1
Total GCC	12,505,515	7621	5166	343	2067	6690	21,887	17.5

GCC, Gulf Cooperation Council; PPC, pediatric palliative care.

## Results

The causes of mortality were similar in all six countries. The top two causes of death were congenital and neonatal diseases, and the least common cause was neoplasia (Table 2).

In 2019, the lowest estimate of the number of children requiring palliative care was just under 22,000, 17.5 for every 10,000 children. All GCC countries had similar ratios individually, with the highest in Oman (21 per 10,000 children) and the lowest in United Arab Emirates (16 per 10,000 children) (Table 3). However, 2.3%–6.6% of children living with LTIs die each year.^31^ Therefore, applying the same ratio to groups for which we were unable to calculate patients with LTIs, we would need to add a minimum of 31,473 children, increasing the estimate of PPC need to 40 per 10,000 children. This may represent a more accurate estimate of the need for PPC, as it considers children *living* with an LTI in addition to those *dying* due to an LTI.

Unlike causes of mortality, we could not identify which types of disease had the most patients needing PPC with certainty. We attempted to estimate the number of children living with LTIs for some conditions such as cerebral palsy and congenital cardiac conditions (Table 1), but this was not possible for other conditions such as genetic diseases, meaning that our findings are highly underestimated. In the absence of robust local (GCC) statistics, global statistics are not helpful for conditions such as genetic diseases, since many genetic conditions are region specific. For example, Sanjad–Sakati syndrome (hypoparathyroidism–retardation–dysmorphism syndrome) and *OSTM1*-related osteopetrosis are almost exclusively seen in GCC countries, and their management is mainly limited to palliation.^32–34^

*Correction added on March 24, 2023 after first online publication of February 16, 2023: In the last row of the Congenital diseases, Neonatal, Infection, Neoplasm, and Other diseases columns the indicated GCC totals were incorrect. The table has been corrected to reflect the proper totals.

## Discussion

To our knowledge, this is the first study attempting to estimate the number of children in the GCC region in need of palliative care based on available mortality data. According to Connor et al,^35^ studies estimating need for adult and PPC based on mortality statistics for chronic incurable illnesses may underestimate the need for palliative care, as children who need palliative care well before the last year of life may not be taken into account.

Congenital conditions are a leading cause of mortality in childhood, and possibly LTI. This may be related to a disproportionately higher incidence of recessive disorders in the Middle East.^36^ In addition, Arabs of the Middle East have a long tradition of consanguineous marriages, and these large family structures leading to unique sociocultural factors must be kept in mind when developing any PPC service in the future.^37^

Our study highlights the unmet need for PPC in the GCC. It could potentially be used as a starting point for advocating for the urgent need to develop PPC services with stakeholders at every level, including governments. Furthermore, medical professionals would recognize that this population of patients exists in their practice and aim to seek learning to fulfill their palliative needs or to become specialists.

Lastly, there is a clear need for further research into the unique characteristics of existing health care systems, sociocultural factors, educational needs, and attitudes of health care workers and available resources that would ideally shape PPC service models in the region.

### Limitations of the study

We relied on secondary databases to determine disease incidence, prevalence, and mortality due to the absence of local pediatric databases. This might affect the accuracy of the need for PPC services. In addition, we looked at available mortality data, which alone is likely to underestimate the need.

## Conclusion

There is significant need for PPC services to meet the medical needs of the pediatric population. Estimating the need for PPC is a critical step toward advocating for PPC services where they are lacking. These data provide medical professionals and policymakers in GCC countries with a first tool to meet the needs of children with LTIs.

## Abbreviations Used

GCC: Gulf Cooperation Council
ICPCN: International Children's Palliative Care Network
IHME: Institute for Health Metrics and Evaluation
LTIs: life-threatening illnesses
PPC: pediatric palliative care
TFSL: Together for Short Lives
WHO: World Health Organization
